# Anterior Chamber Measurements in Healthy Children: A Cross-Sectional Study Using Optical Coherence Tomography

**DOI:** 10.1167/tvst.10.6.13

**Published:** 2021-05-07

**Authors:** Budor S. A. Edawaji, Irene Gottlob, Frank A. Proudlock

**Affiliations:** 1 University of Leicester Ulverscroft Eye Unit, Department of Neuroscience, Psychology and Behaviour, University of Leicester, Leicester, UK

**Keywords:** trabecular meshwork, angle opening distance, anterior chamber width, Schwalbe's line, scleral spur

## Abstract

**Purpose:**

To establish anterior chamber measurements in children and investigate the influence of demographic factors on anterior chamber development.

**Methods:**

Handheld optical coherence tomography was used to scan the anterior chamber of participants’ eyes, without sedation. ImageJ was used to generate quantitative anterior chamber measurements, including central corneal thickness (CCT), anterior chamber width, trabecular meshwork length (TML), Schwalbe's line–angle opening distance (SL-AOD), and trabecular iris surface area (SL-TISA). The average anterior chamber measurements per age group, with 95% prediction intervals, were estimated using fractional polynomial modeling. Mixed regression models were used to evaluate the influence of age, gender, eye, angle, and refractive error variation on anterior chamber measurements.

**Results:**

Scans from 223 healthy children (2 days to 15 years of age) and 59 adults (16 to 47 years of age) were included. The anterior chamber width, TML, Schwalbe's line–angle opening distance, and Schwalbe's line–trabecular iris surface area significantly increased, whereas CCT decreased with aging (all *P* < 0.001). The anterior chamber has a rapid phase of development during the first 18 months of age and reaches maturity by the age of 5 years. Girls have significantly smaller anterior chambers compared with boys (all *P* < 0.001). There was no difference between right and left eye development (all *P* > 0.05). The temporal TML development was significantly greater than the nasal TML (*P* < 0.05). CCT development was negatively correlated with refractive power.

**Conclusions:**

This novel, non-invasive study describes the postnatal development of anterior chamber in newborn children.

**Translational Relevance:**

Our established quantitative measurements have potential clinical use in understanding anterior segment diseases.

## Introduction

The morphometry of the anterior chamber in adults has been well documented in relation to demographic factors such as age, gender, and ethnicity, in addition to refractive error,^1^^,^^2^ and it has clinical implications. For example, closed-angle glaucoma is more common in women due to a narrower anterior chamber as compared with men.^3^^,^^4^ Closed-angle glaucoma prevalence is higher in individuals of Chinese ethnicity,^5^ who have shallower anterior chambers compared with Caucasians.^6^^,^^7^ The success of corrective refractive surgery is dependent on a precise preoperative evaluation of refraction and ocular biometry.^8^ Anterior chamber angle geometry is important for the management of anterior segment diseases, such as glaucoma.^9^

To date, there has been scarce information about anterior chamber geometry in children. It has been reported, through the use of magnetic resonance imaging, that the axial length and anterior chamber depth increase with age.^10^ Reports show that lens thickness decreases in early childhood (≥6 years)^11^ and then slightly increases in late childhood.^12^ Also, the central corneal thickness becomes thinner with increasing age in children.^13^

Ultrasound biomicroscopy has been used to establish normative quantitative anterior chamber measurements in children (1–60 months old), which show a positive linear correlation between the anterior chamber depth and angle opening distance with age.^14^ However, ultrasound biomicroscopy requires the instillation of local anesthesia and direct contact of the ultrasound biomicroscopy probe to the cornea. This makes pediatric examination difficult and may affect the accuracy of the anterior chamber angle measurements. In contrast, anterior segment optical coherence tomography (AS-OCT) imaging has the advantage of being non-invasive, with no direct eye contact, while providing high-resolution imaging.

We used handheld OCT to acquire three-dimensional images of the anterior segment, in a large cohort of normal children, without sedation, to investigate the postnatal normal development of the anterior chamber. Our focus was on the early years of life, when most of the rapid changes in development take place, based on histological studies.^15^^,^^16^ We also aimed to identify when maturity of the anterior chamber is reached. The data obtained form the basis of normative anterior chamber measurements for children, which can be used clinically for diagnostic purposes and tracking eye disease progression. We also investigated the impact of factors such as gender, eye, and refractive differences.

## Methods

The study cohort included 223 full-term infants and children (104 females and 119 males), ages 2 days to 15 years (mean, 5.7 ± 4.1 years). Newborns were prospectively recruited from the Maternity Unit at the University Hospitals of Leicester, Leicester, UK. Older children were recruited from Leicester City nurseries and schools. Adult values were established from an additional 59 adults (41 females and 18 males), ages 16 to 47 years (mean, 33.5 ± 8.7 years). Adult participants were students and staff of the University of Leicester. This study followed the tenets of the Declaration of Helsinki and was approved by the local ethics committee. Informed consent was obtained from all participant parents or guardians and older children gave their assent before examination.

All subjects underwent an examination of best-corrected visual acuity (BCVA), ocular motility, stereopsis, refraction, slit-lamp biomicroscopy, fundoscopy, and posterior and AS-OCT. Refraction was tested in most children using a portable Plusoptix A12C autorefractor (Plusoptix, Nuremberg, Germany), without cycloplegic eye drop administration. The BCVA of infants and young children unable to cooperate was tested using preferential looking (Teller Acuity Cards; Washington Research Foundation, Seattle, WA). If cooperation did not allow us to obtain BCVA or refraction, children were invited back at an older age for re-examination; logMAR Kay acuity cards were used for cooperative children. All subjects were free of ocular and neurological pathology and had good general health.

**Figure 1.**
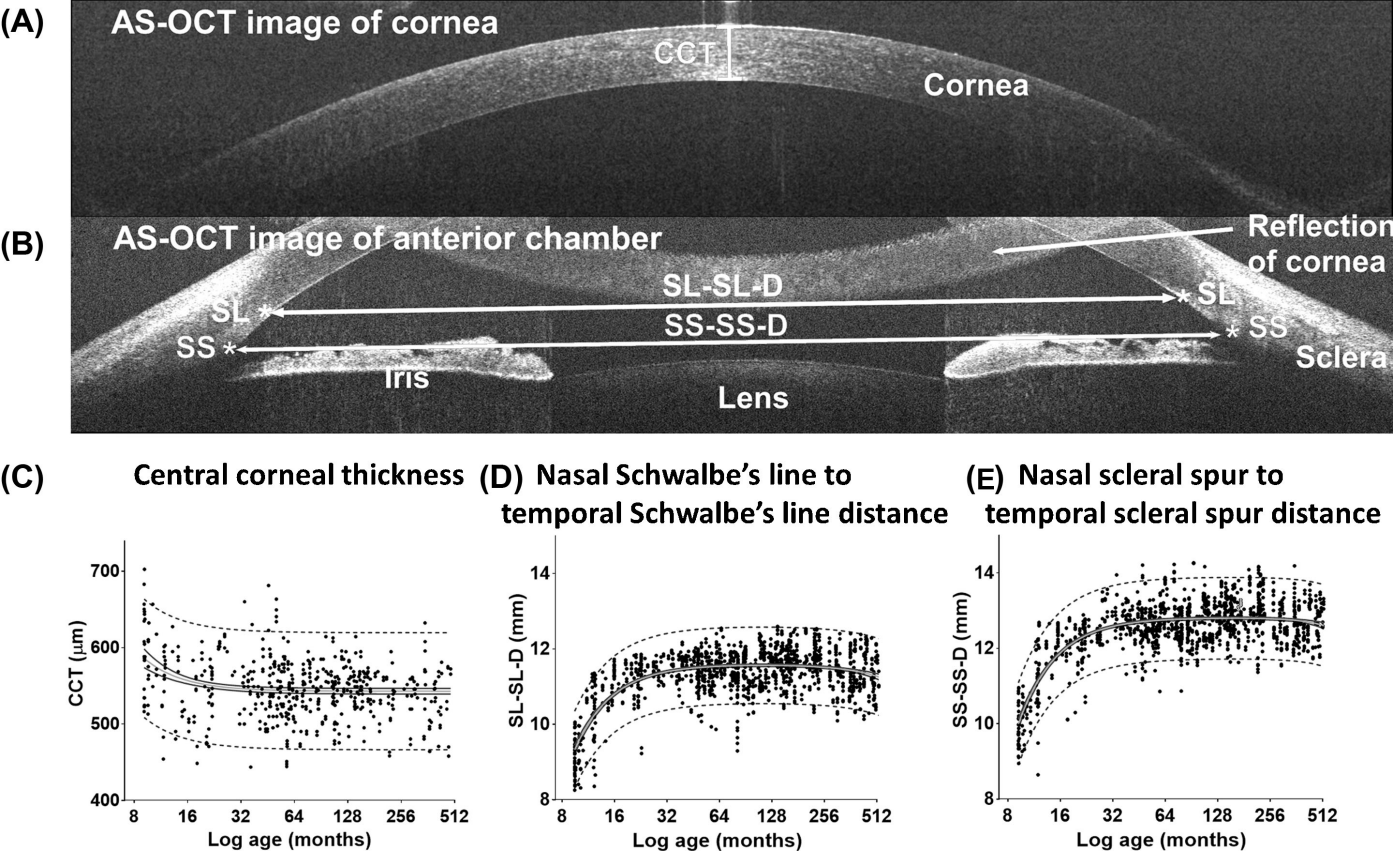
Anterior chamber OCT tomograms and scatterplots showing the development of anterior chamber width and central corneal thickness (CCT). (**A**) B-scan showing the measurement of CCT, (**B**) measurements of anterior chamber width, and (**C**) development of CCT, nasal Schwalbe's line to temporal Schwalbe's line distance (SL-SL-D) and nasal scleral spur to temporal scleral spur distance (SS-SS-D), with postmenstrual age (PMA) in months. Mean value (*gray* *lines*), 95% confidence intervals of mean (*black lines*), 95% prediction intervals (*dashed lines*), and individual measurements (*black dots*) are shown.

All subject eyes were scanned with an Envisu C-Class handheld OCT system (Leica Microsystems, Wetzlar, Germany) without sedation. The protocol used had a volumetric horizontal scan of 18 mm width and 6 mm height and contained 11 B-scans, with 3000 A-scans per B-scan. The rapid acquisition sequence used (0.96-second duration for the full scan and 87 ms for each B-scan) helped reduce motion artifacts. This was also aided by the child's head being kept still by the parent/carer and with the child's attention being distracted by watching a cartoon video. The scans were acquired and reviewed by a trained examiner. The criteria defined for the quality of usable images included (1) both temporal and nasal angles could clearly be seen on the horizontal B-scan; (2) a clear peripheral cornea was visible; and (3) both irises and corneoscleral junctions could be distinguished. The examination room light was controlled at ∼200 lux*.* Limited scanning depth of the handheld OCT (only 2.5 mm in tissue) does not permit imaging of the whole anterior segment; therefore, we obtained two separate scans: one scan showing the cornea (Fig. 1A) and another scan showing the nasal and temporal anterior chamber angles (Fig. 1B).

The OCT images were imported into ImageJ software (National Institutes of Health, Bethesda, MD)^17^ for analysis. The best individual B-scans were selected to further assess the quality of images and perform quantitative measurements. At least one good quality scan of the anterior chamber and one of the cornea was successfully obtained per eye, per participant.

The central corneal thickness (CCT) was measured from a separate corneal image (Fig. 1A). The anterior chamber measurements were obtained from the B-scan showing both nasal and temporal angles (Fig. 1B). An ImageJ script was developed to perform semiautomated measurements after manual identification of the angle landmarks: the scleral spur (SS) and Schwalbe's line (SL) in both nasal and temporal angles as described by Sakata et al.^18^ The agreement in our identification of SS and SL was tested by two assessors. The intra-assessor and inter-assessor agreement yielded high repeatability and reproducibility of the localization of SS and SL.^19^

**Figure 2.**
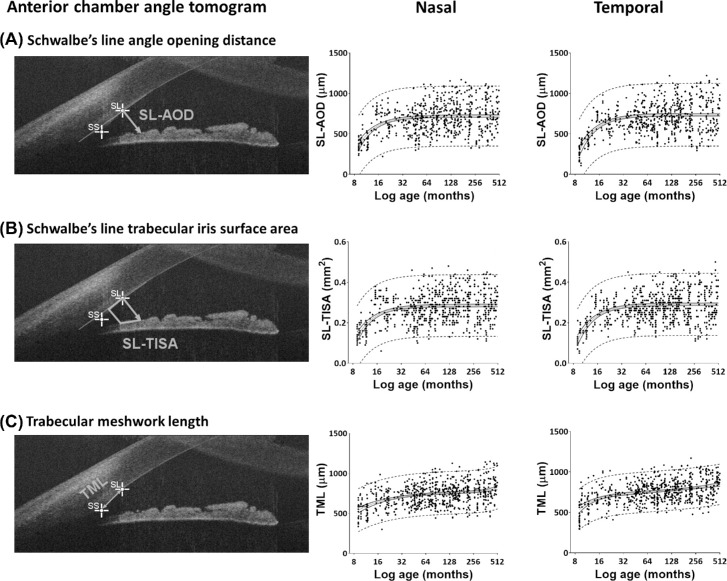
Anterior chamber angle OCT tomogram and scatterplots showing the development of nasal and temporal anterior chamber angle measurements. (**A**) SL-AOD, (**B**) SL-TISA, and (**C**) TML, with PMA in months. Mean value (*gray* *lines*), 95% confidence intervals of mean (*black lines*), 95% prediction intervals (*dashed lines*), and individual measurements (*black dots*) are shown for nasal and temporal angles separately.

The anterior chamber width (ACW) was measured as the distance between (1) the nasal scleral spur to temporal scleral spur (SS-SS-D), and (2) the nasal Schwalbe's line to temporal Schwalbe's line (SL-SL-D). The nasal and temporal angle parameters were derived from the Schwalbe's line in each angle. These measurements correspond to Schwalbe's line–angle opening distance (SL-AOD) (Fig. 2) and Schwalbe's line–trabecular iris surface area (SL-TISA), described by Cheung et al.^20^ The trabecular meshwork length (TML) was measured as the distance between Schwalbe's line and the scleral spur. We investigated the test–retest reproducibility of these measurements and detected high reproducibility and repeatability, with intraclass correlation coefficient > 0.9 and small measurement errors.^21^

Multivariable fractional polynomial regression models were used to model the effect of age on anterior segment measurements. The modeling automatically transformed age into a set of powers (–2, –1, –0.5, 2, or 3 or logarithmic transformations) in order to achieve a more normal distribution of the skewed data. Linear mixed models (adjusted for the transformed age, eye laterality, and gender variation) were then used to establish normative anterior chamber measurements by calculating the mean fit, 95% confidence intervals, and 95% upper and lower prediction intervals of the data. The nasal and temporal angle measurements were analyzed separately. We also calculated the rate of change of each measurement, per year, to determine the development of the anterior chamber and to identify the age when the maximum value, or a plateau of adult values, is reached. Gender differences in anterior segment measurements and difference between nasal and temporal angle measurements were assessed at different ages, including at birth and at 1, 5, 18, and 35 years of age.

The correlation between spherical equivalent and the anterior chamber measurements was investigated in a subgroup of 154 children (average spherical equivalent power = 0.5 ± 1.1 diopters). First-order partial correlations were used to adjust for the effect of age, gender, eye, and angle variations. The analysis was performed using Stata 15 (StataCorp, College Station, TX).

## Results

A total of 1464 anterior chamber measurements (48% from left eyes and 52% from right eyes) and 574 central corneal thickness measurements from 282 participants at different ages were included in this study. Only high-quality images with a clear cornea and clear corneoscleral junction structures of both temporal and nasal angles were included. For this reason, in 84 participants the anterior chamber image from only one eye was included. The demographics of the children and adults, categorized per age group, are summarized in Supplementary Table S1. The established mean values and 95% prediction intervals of central corneal thickness, ACW (SS-SS-D and SL-SL-D), and nasal and temporal angle measurements (TML, SL-AOD, and SL-TISA), per age group, are presented in Tables 1 and 2.

**Table 1.** Established Normative Measurements of the ACW and CCT Categorized by Age Group

	Mean (Lower and Upper 95% Prediction Intervals)		
	ACW (mm)		
Age	SL-SL-D	SS-SS-D	CCT (µm)
<1 mo	9.44 (8.42–10.46)	10.13 (9.05–11.22)	593.7 (503.7–663.3)
1.1–6 mo	10.31 (9.29–11.32)	11.21 (10.13–12.30)	566.5 (482.2–654.3)
6.1–12 mo	10.99 (9.98–12.01)	12.07 (10.98–13.15)	553.2 (474.2–634.5)
1.1–2 y	11.38 (10.36–12.39)	12.54 (11.46–13.62)	546.4 (469.5–623.7)
2.1–3 y	11.46 (10.45–12.48)	12.65 (11.57–13.73)	545.1 (467.9–622.5)
3.1–4 y	11.51 (10.50–12.53)	12.71 (11.63–13.80)	544.1 (467.3–621.0)
4.1–5 y	11.53 (10.52–12.55)	12.74 (11.66–13.83)	543.7 (466.9–620.4)
5.1–6 y	11.55 (10.53–12.56)	12.76 (11.68–13.85)	543.4 (466.7–620.0)
6.1–8 y	11.56 (10.54–12.57)	12.78 (11.69–13.86)	543.2 (466.5–619.8)
8.1–10 y	11.56 (10.54–12.57)	12.79 (11.70–13.87)	543.0 (466.4–619.6)
10.1–14 y	11.55 (10.54–12.56)	12.79 (11.71–13.87)	542.9 (466.3–619.5)
14.1–18 y	11.53 (10.51–12.54)	12.79 (11.70–13.87)	542.8 (466.2–619.4)
18.1–25 y	11.49 (10.47–12.50)	12.77 (11.69–13.85)	542.8 (466.2–619.4)
25.1–35 y	11.40 (10.39–12.42)	12.72 (11.64–13.80)	542.7 (466.2–619.3)
35.1–45 y	11.27 (10.26–12.29)	12.62 (11.54–13.71)	542.7 (466.2–619.3)
>45 y	11.09 (10.07–12.11)	12.44 (11.34–13.53)	542.7 (466.2–619.3)

**Table 2.** Established Normative Measurements of Nasal and Temporal Anterior Chamber Angle Measurements Categorized by Age Group

	Anterior Chamber Angle Measurements, Mean (Lower and Upper 95% Prediction Intervals)					
	Temporal			Nasal		
Age	TML (µm)	SL-AOD (µm)	SL-TISA (mm^2^)	TML (µm)	SL-AOD (µm)	SL-TISA (mm^2^)
<1 mo	547.23 (295.77–798.69)	311.05 (–81.18 to 703.28)	0.12 (–0.03 to 0.28)	543.61 (275.64–811.59)	369.34 (–5.8 to 743.93)	0.14 (–0.02 to 0.29)
1.1–6 mo	623.40 (373.95–872.86)	485.62 (96.34–874.90)	0.19 (0.04–0.35)	590.99 (323.89–858.09)	498.03 (125.8–870.18)	0.19 (0.04–0.34)
6.1–12 mo	683.14 (434.28–932.00)	617.05 (228.90,1005.20)	0.24 (0.09–0.40)	647.12 (380.73–913.52)	614.27 (243.8–985.21)	0.24 (0.09–0.39)
1.1–2 y	722.43 (473.61–971.26)	693.21 (305.26–1081.16)	0.27 (0.12–0.43)	693.44 (427.33–959.54)	678.59 (307.8–1049.33)	0.27 (0.02–0.42)
2.1–3 y	735.63 (486.82–984.44)	711.97 (324.01–1099.93)	0.28 (0.13–0.43)	713.17 (447.10–979.24)	696.60 (325.8–1067.34)	0.28 (0.02–0.43)
3.1–4 y	745.09 (496.31–993.87)	722.17 (334.20–1110.15)	0.29 (0.13–0.44)	726.76 (460.69–992.83)	706.17 (335.8–1076.93)	0.28 (0.03–0.43)
4.1–5 y	751.66 (502.92–1000.40)	727.00 (339.02–1114.98)	0.29 (0.13–0.44)	733.84 (467.77–999.92)	710.13 (339.8–1080.89)	0.28 (0.03–0.43)
5.1–6 y	758.17 (509.47–1006.88)	730.28 (342.29–1118.26)	0.29 (0.13–0.44)	739.63 (473.54–1005.72)	712.80 (342.8–1083.56)	0.28 (0.03–0.43)
6.1–8 y	764.44 (515.77–1013.11)	732.31 (344.32–1120.30)	0.29 (0.14–0.44)	745.85 (479.74–1011.95)	715.20 (344.8–1085.97)	0.28 (0.03–0.44)
8.1–10 y	772.12 (523.49–1020.76)	733.95 (345.96–1121.95)	0.29 (0.14–0.44)	751.36 (485.24–1017.49)	716.86 (346.8–1087.64)	0.28 (0.03–0.44)
10.1–14 y	781.92 (533.31–1030.54)	735.11 (347.11–1123.11)	0.29 (0.14–0.44)	756.96 (490.83–1023.10)	718.06 (347.8–1088.84)	0.28 (0.03–0.44)
14.1–18 y	795.78 (547.13–1044.43)	735.94 (347.94–1123.94)	0.29 (0.14–0.44)	762.83 (496.69–1028.97)	718.83 (348.8–1089.61)	0.28 (0.03–0.44)
18.1–25 y	808.29 (559.54–1057.04)	736.28 (348.28–1124.28)	0.29 (0.14–0.44)	768.61 (502.49–1034.74)	719.18 (348.8–1089.97)	0.29 (0.03–0.44)
25.1–35 y	826.04 (577.06–1075.03)	736.53 (348.53–1124.54)	0.29 (0.14–0.44)	781.58 (515.43–1047.73)	719.45 (348.8–1090.23)	0.29 (0.03–0.44)
35.1–45 y	842.84 (593.52–1092.17)	736.65 (348.64–1124.65)	0.29 (0.14–0.44)	800.98 (534.48–1067.49)	719.55 (348.8–1090.34)	0.29 (0.03–0.44)
>45 y	858.78 (609.03–1108.54)	736.70 (348.70–1124.71)	0.29 (0.14–0.44)	819.09 (551.88–1086.29)	719.59 (348.8–1090.38)	0.29 (0.03–0.44)

### Postnatal Development of Anterior Chamber

The effects of aging on ACW (SS-SS-D and SL-SL-D) and central corneal thickness are shown in Figure 1C. The effects of aging on nasal and temporal angle measurements (TML, SL-AOD, and SL-TISA) are shown in Figure 2. The best curve fit of all measured parameters shows a nonlinear relationship with age, indicating that the greatest rate of postnatal development of the anterior chamber occurs earlier in life. There was a small reduction of CCT with increasing age. CCT decreased by 40 µm (7%) during the first year of age (*F* = 2.90, *P* = 0.03) and reached a plateau by approximately 3 years of age. The ACW and anterior chamber angle measurements both increased significantly with increasing age. SS-SS-D and SL-SL-D increased by 19% and 16%, respectively, from birth during the first year of age (*F* = 73.44, *P* < 0.001) and reached maximum levels by 4 years of age.

There was significant widening of nasal and temporal anterior chamber angles during the first year of age (*F* = 63.82 for SL-AOD, *F* = 60.04 for SL-TISA; all *P* < 0.001). SL-AOD and SL-TISA increased by 66% and 77% in the nasal angle and by 98% and 99% in the temporal angle, respectively, during the first year of age. This widening slowed down throughout childhood and reached a plateau by the age of 5 years. The TMLs became elongated by 19% and 25%, respectively (*F* = 11.50, *P* < 0.001). The developmental trajectories of trabecular meshwork showed continuous slight elongation of trabecular meshwork up to adulthood.

### Impact of Demographic Factors on Anterior Chamber Parameters

**Figure 3.**
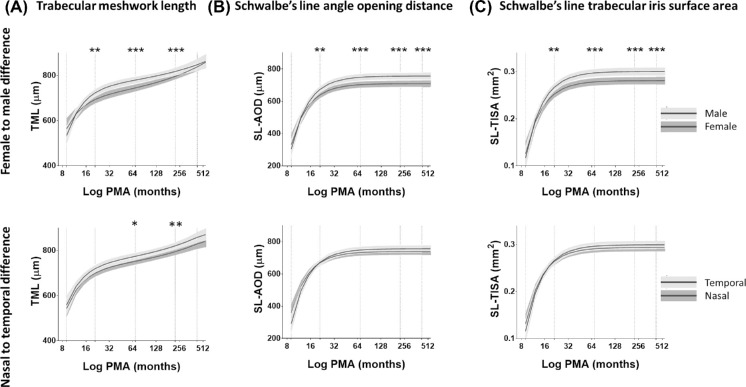
Polynomial plots comparing the development of anterior chamber angle measurements of males and females and between nasal and temporal angle. (**A**) TML (in µm), (**B**) SL-AOD (in µm), and (**C**) SL-TISA(in mm^2^). Mean curve fit (*black lines*) and 95% confidence intervals of each curve fit (*gray* *areas*) are shown. The differences in anterior chamber angle measurements at different ages (*vertical dashed line*) are indicated with asterisks: ^*^P < 0.05, ^**^P < 0.01, ^***^P < 0.001.

A significant gender difference in the anterior chamber parameters, after controlling for the effect of age, was detected in the mixed models (all *P* < 0.05). Females had a shorter ACW (SS-SS-D and SL-SL-D) compared with males. The increase in the male TML was statistically greater than in the female TML. There was a significantly narrower angle width (SL-AOD and SL-TISA) in females compared with males (Fig. 3A). No significant gender differences in CCT were detected (*P* > 0.05). There were no significant differences between the right and left eyes for all measurements at any age (all *P* > 0.05).

### Variation of Nasal and Temporal Trabecular Meshwork Development

The difference between nasal and temporal parameters at different age points, including at birth and at 1, 5, 18, and 35 years of age, revealed that the amount of TML elongation was significantly greater at the temporal angle compared with the nasal angle (Fig. 3B). The nasal and temporal TMLs were not different at birth (*P* = 0.59). However, at the age of 5 years, the rate of elongation of the temporal TML was significantly higher compared with the increase in nasal TML (*P* < 0.05). This difference continued to be significant at the age of 18 years (*P* < 0.01) but was insignificant at the age of 35 years (*P* > 0.05). The temporal and nasal TMLs increased by 45% and 40%, respectively, for the first 18 years of age. This indicated nasal–temporal asymmetry in the development of TML during childhood and early adulthood. No difference was detected between the nasal and temporal angle width measurements (*P* > 0.05).

### Correlation Between Anterior Chamber Measurements and Refractive Error

Spherical refractive errors in a subgroup of 154 children (6 months to 15.4 years of age; 44% males and 56% females) were compared with the anterior chamber and corneal thickness measurements. This included 800 anterior chamber angle images (414 from right eyes, 52%; 386 from left eyes, 48%) and 289 corneal images (143 from right eyes, 49%; 146 from left eyes, 51%). The results are summarized in Supplementary Table S2. After adjusting for the effect of age, there was a weak but significant negative correlation between spherical refractive power and angle width measurements (SL-AOD Pearson correlation coefficient *r* = –0.26 and SL-TISA *r* = –0.24; all *P* < 0.001). The findings indicate that myopic eyes had wider angles and the hypermetropic eyes had shallower angles. There was a significant negative correlation between spherical refractive power and CCT (*r* = –0.13, *P* < 0.01). Therefore, a thick cornea was associated with myopia, and a thin cornea was associated with hypermetropia. In contrast, the ACW had a weak positive correlation with spherical power (SS-SS-d *r* = 0.10, SL-SL-D *r* = 0.08; both *P* < 0.05). This suggests that the increase of ACW with age is associated with a weak hypermetropic shift.

## Discussion

Histological studies have previously revealed that the development of the anterior chamber continues postnatally in both humans^15^^,^^16^ and mice.^22^^,^^23^ Reme et al.^16^ studied five enucleated eyes of fetuses and infants and reported that the anterior chamber angle reached the configuration of the adult eye by 1 to 4 years after birth, histologically, and the final cellular and extracellular maturation of trabecular meshwork was achieved by 1 to 8 years of age. To the best of our knowledge, we present for the first time normative measurements of the anterior chamber in children from birth using AS-OCT. Our study shows that the anterior chamber grows rapidly during the first year of life and reaches maturity approximately by the age of 5 years. Unlike the histology specimens, which were manipulated by chemicals and derived from a small sample size, we had the advantage of analyzing in vivo OCT measurements of a large sample size of healthy children with the maintenance of tissue geometry. During postnatal development, the trabecular meshwork became elongated; the nasal and temporal angle width doubled in size during the first year of life and reached adult values by the age of 5 years. The trabecular meshwork continued to elongate posteriorly up to adulthood at a slow rate but without further widening of angle width. Consistently, we observed that the ACW increased and then stabilized by the age of 3 years.

The trend of anterior chamber changes with age are similar to the development of axial length,^24^ optic nerve,^25^ and fovea.^26^ Using A-scan ultrasonography, the axial length has been reported to increase rapidly during the first 18 months, from about 16.8 mm at birth to 20 mm at 1 year (an increase of about 19%, similar to our rate of ACW development) and to 21 mm at 4 years of age.^24^ Using magnetic resonance imaging, Munro et al.^10^ observed that the anterior chamber depth increased and reached a plateau by 2 years of age, whereas axial length continued to increase at a slower rate up to 20 years of age. A continuous increase in axial length (by 0.09 mm per year until the age of 18 years) was also observed, through the use of a LENSTAR Biometer (Haag-Streit, Köniz, Switzerland) among Iranian students 6 to 18 years of age.^11^ In addition, iris bowing has been reported to play a crucial role in the size of the anterior chamber angle width.^27^^,^^28^ Sng et al.^29^ reported that forward iris curvature and iris area increase with age and that forward iris curvature is associated with narrowing of the anterior chamber angle. We did not measure the iris thickness, but we observed that at 2 days the iris was thin and flat with no visible crypts; however, by the age of 5 years, the configuration of the anterior chamber was similar to that in adults, with backward bowing of the pupillary iris and well-formed visible iris crypts. We did not detect any narrowing of either the nasal or temporal angle during adulthood. This is in agreement with a recent study of Chinese children (6 to 18 years old), which used a Pentacam Scheimpflug camera (Oculus, Wetzlar, Germany) and found that the anterior chamber angle degree was stable in this age group.^13^

### Gender Variation of Anterior Chamber Parameters

Significant gender variations in anterior chamber parameters in adults have been documented using AS-OCT. Women have narrower anterior chamber angles,^30^ shallower anterior chamber depths, shorter anterior chamber widths, and thicker lenses as compared with men.^5^ Significantly smaller anterior chamber depths, corneal diameters, and axial lengths have been reported in girls between 6 to 18 years of age compared with boys.^31^ Girls also have thicker lens compared with boys,^11^ and axial length development occurs at a slower rate in girls compared with boys.^32^ Similar to these studies, we detected shorter anterior chamber widths and narrower anterior chamber angles in females compared with males from the age of 1 year and continuing throughout childhood into adulthood. Previous AS-OCT studies have failed to detect gender variations in trabecular meshwork measurements in adults^33^^,^^34^ and children (3 to 18 years old).^35^ However, an association of glaucoma with shorter trabecular meshwork has been reported.^34^^,^^36^ We found that the trabecular meshwork became longer in males compared with females. This might explain why closed-angle glaucoma is more common among women, as the shorter trabecular meshwork in females is more likely to become blocked by the iris.

### Limitations of Study

Anterior chamber development is not easy to interpret without taking into account the development of axial length with the lens and the iris. Many of the developmental changes in the anterior and posterior segments of the eye are likely to be due to significant changes in axial length that take place in early years and were not measured in this study. The measurement of axial length in young children is extremely challenging at present, although the new generation of OCT devices may be able to incorporate this measurement, thus offering even better clinical utility. We also did not measure anterior chamber depth, lens thickness, iris curvature, and area due to the limited depth of spectral-domain OCT imaging. The analysis was based on manual identification of anterior chamber landmarks, which could cause measurement errors. Also, we studied only nasal and temporal meridians and avoided pulling the eyelid to expose the superior meridian.

### Clinical Impact of Understanding the Normal Development of the Anterior Chamber

Having knowledge of the normal development of the anterior chamber could play a potential role in enabling the detection and evaluation of anterior chamber pathology. It will also help us to understand, diagnose, and surgically manage pediatric ocular diseases such as congenital glaucoma, cataract, and anterior segment dysgenesis.^37^ Several studies have addressed the mechanisms of congenital glaucoma by investigating the abnormal development of the anterior chamber in vitro or using mice models. Comparing the anterior chamber measurements of infantile glaucoma with our normative values could improve our understanding of the disease progression and its management.

The success of cataract and refractive surgery depends on accurate ocular biometry. It is not possible to accurately predict the correct power for implanted intraocular lens for use in infants after congenital cataract surgery, due to rapid eye growth during the first few years of life and the unpredictable postoperative myopic shift.^38^^,^^39^ We detected a significant correlation between anterior chamber measurements and myopic shift. Knowledge of eye maturation and understanding its correlation with refraction are likely to help in determining the best time to perform corrective refractive surgery in children with larger refractive errors. Hence, investigations of patients with refractive errors in comparison with normative data would be important.

## Conclusions

We have presented for the first time, to the best of our knowledge, normative measurements, with 95% prediction limits, of anterior chamber width, trabecular meshwork length, anterior chamber angle width, and central corneal thickness obtained using handheld OCT. We determined the trajectories of anterior chamber development from birth to adulthood, and our established normative measurements have the potential to improve management of anterior segment pathology.

## Supplementary Material

Supplement 1

Supplement 2
