# Supplementary material for: Anterior Chamber Measurements in Healthy Children: A Cross-Sectional Study Using Optical Coherence Tomography
Source: Transl Vis Sci Technol. 2021 May 7;10(6):13. doi: 10.1167/tvst.10.6.13 (PMC8114001; doi:10.1167/tvst.10.6.13)
Supplement: Supplement 1 [file tvst-10-6-13_s001.pdf]

***Table S1:*** *Demographics of healthy participants categorized by age group and the hand-held optical coherence tomography measurements per eye and angle.*

| Age group | Number | Mean ± SD | Minimum | Maximum |
|---|---|---|---|---|
| < 1 week | 11 | 2.30 ± 1.50 days | 1 day | 4 days |
| 1 week - 0.9 month | 7 | 17.80 ± 3.10 days | 15 days | 23 days |
| 1 - 5.9 months | 12 | 3.00 ± 1.50 | 0.80 | 5.80 |
| 6 - 11.9 months | 11 | 9.10 ± 1.90 | 6.20 | 11.90 |
| 1 - 1.9 years | 17 | 1.40 ± 0.30 | 1.03 | 1.96 |
| 2 - 2.9 years | 20 | 2.33 ± 0.23 | 2.00 | 2.73 |
| 3 - 3.9 years | 29 | 3.44 ± 0.29 | 3.04 | 3.92 |
| 4 - 4.5 years | 18 | 4.34 ± 0.26 | 4.01 | 4.96 |
| 5 - 5.9 years | 17 | 5.40 ± 0.26 | 5.02 | 5.77 |
| 6 - 6.9 years | 18 | 6.47 ± 0.28 | 6.01 | 6.96 |
| 7 - 8.9 years | 20 | 8.01 ± 0.46 | 7.18 | 8.72 |
| 9 - 11.9 years | 24 | 10.57 ± 0.90 | 9.03 | 11.91 |
| 12 - 15.9 years | 19 | 13.84 ± 1.13 | 12.06 | 15.43 |
| 16 - 19.9 years | 15 | 17.55 ± 0.67 | 16.09 | 18.67 |
| ≥ 20 years | 44 | 33.19 ± 7.41 | 21.11 | 47.06 |
| Total | 282 | | | |
| **Ethnicity** | | | **Number (%)** | |
| White European | | | 180 (64) | |
| White North African | | | 41 (15) | |
| Indian | | | 47 (17) | |
| Mixed ethnicity | | | 14 (5) | |
| **Gender** | | | **Number** | |
| Female | | | 145 (51%) | |
| Male | | | 137 (49%) | |
| **Anterior chamber angle images (n = 1464)** | | | | |
| **Left eyes (number, %)** | | | **Right eyes (number, %)** | |
| 702 (48%) | | | 762 (52%) | |
| Temporal | Nasal | | Temporal | Nasal |
| 350 (24%) | 352 (24%) | | 369 (25%) | 393 (27%) |
| **Corneal images (n = 574)** | | | | |
| **Left eye (number, %)** | | | **Right eye (number, %)** | |
| 287 (50%) | | | 287 (50%) | |
