# Supplementary material for: Anterior Chamber Measurements in Healthy Children: A Cross-Sectional Study Using Optical Coherence Tomography
Source: Transl Vis Sci Technol. 2021 May 7;10(6):13. doi: 10.1167/tvst.10.6.13 (PMC8114001; doi:10.1167/tvst.10.6.13)
Supplement: Supplement 2 [file tvst-10-6-13_s002.pdf]

Table S2: *First order partial correlation between anterior chamber measurements and spherical power.*

		Anterior chamber angle measurements			Anterior chamber width		Central corneal thickness
		**TML**	**SL-AOD**	**SL-TISA**	**SS-SS-D**	**SL-SL-D**	**CCT**
		n = 800					n = 289
**Spherical power**	**r**	0.02	-0.26	-0.24	0.10	0.08	-0.13
	**β**	2.38	-43.25	-0.02	0.05	0.03	-4.63
	***p* value**	Ns	**<0.0001**	**<0.0001**	**0.004**	**0.03**	**0.002**

TML = trabecular meshwork length SL-AOD = Schwalbe's line angle opening distance. SL-TISA = Schwalbe's line trabecular iris surface area. ACW = anterior chamber width; SS-SS-D = nasal scleral spur to temporal scleral spur distance. SL-SL-D = nasal Schwalbe's line to temporal Schwalbe's line distance. CCT = central corneal thickness. Pearson correlation coefficient (r), coefficient of difference (β). The effecct of age was adjusted, n = number of images, Ns = non-significant difference.
